# Supplementary material for: Are carbon emissions trading and green financial instruments synergistic? -Comprehensive quantitative research based on content analysis
Source: PLoS One. 2024 Mar 7;19(3):e0298601. doi: 10.1371/journal.pone.0298601 (PMC10919601; doi:10.1371/journal.pone.0298601)
Supplement: S1 Data — (ZIP) [file pone.0298601.s001.zip › Data/地级市Z_F工作报告.zip!/┴╔─■/║∙┬1⁄2╡║╩╨/└┤╫╘╣1⁄2╓┌║┼í╛┬φ┐╦╩2╛▌═°í┐_2011.docx]

各位代表：

　　现在，我代表市人民政府向大会报告工作，请予审议，并请市政协各位委员和列席会议的同志提出意见。

一、“十一五”时期回顾和2010年工作

　　“十一五”是我市经济社会发展取得显著成绩的五年。在市委的正确领导下，在市人大、市政协的监督支持下，市政府认真贯彻落实科学发展观，抢抓“东北振兴和沿海开放”的双重机遇，解放思想，攻坚克难，全面完成“十一五”规划确定的各项目标任务。

　　“十一五”期间，我市拉开了沿海经济带开发建设大会战的序幕。“主题概念”产业园区从无到有，由小变大，达到12个，规划面积379平方公里，其中100平方公里实现“七通一平”，已经成为全市对外开放的先导区和特色产业聚集区。

　　“十一五”期间，我市固定资产投资累计1250亿元，是建市以来前16年总和的1.7倍，“项目牵动”战略取得丰硕成果。

　　“十一五”期间，我市有22户国企完成改制，妥善安置6万多名职工，国有企业的历史性难题得到有效化解。

　　“十一五”期间，我市免除农业税赋13亿元；支农投入39.5亿元，是“十五”的5.8倍；建立了“新农合”制度，取消了农村义务工制度，“三农”工作成效显著。

　　“十一五”期间，我市实施建市以来最大规模的棚户区改造，9.3万居民喜迁新居，告别了“行路难、吃水难、如厕难”等生活诸难的历史。

　　“十一五”期间，葫芦岛港口岸扩大开放通过国家验收，标志着我市对外开放跃上新台阶，全市人民期盼全面开放的夙愿，终于变成现实！

　　各位代表，“十一五”的成绩来之不易，是在历届班子打下的良好基础上，全市人民用饱满的发展激情，战胜了一个又一个困难，推动经济社会实现了历史性新跨越。我们可以自豪地说，“十一五”是我市经济实力提升最快、城乡面貌变化最大、群众得到实惠最多的五年！

　　各位代表，刚刚过去的2010年，是实施“十一五”规划的收官之年，也是本届政府的开局之年。在这承前启后的关键时期，我市主要经济指标均以两位数或三位数的速度增长，全面完成了市四届人大一次会议确定的各项目标任务。

　　预计2010年，全市地区生产总值531.4亿元，同比增长15.5%，是2005年的1.7倍。地方财政一般预算收入55.9亿元，同比增长27.4%，是2005年的2.5倍。全社会固定资产投资500亿元，同比增长57.7%，是2005年的5倍。社会消费品零售总额233.2亿元，同比增长18.7%，是2005年的2.3倍。城镇居民人均可支配收入17371元，同比增长13.5%，是2005年的2倍。农民人均纯收入6597元，同比增长17.9%，是2005年的1.9倍。

　　1、沿海经济带建设日新月异，开放型经济取得丰硕成果

　　抢抓辽宁沿海经济带上升为国家战略的重大历史机遇，明确重点产业园区为市政府派出机构，实行市级领导包保责任制，加速了产业集聚步伐。12个“主题概念”产业园区建成和在建项目375个、总投资近1000亿元，完成固定资产投资300亿元、同比增长225%，已经成为经济增长的强大引擎。

　　坚持“走出去、请进来”，全市实际利用外资2.2亿美元，同比增长199%，是2005年的9倍；引进省外资金126亿元，同比增长51.8%，是2005年的13倍。外贸进出口总额14亿美元，同比增长38%。

　　2、一批重大项目开工建设，产业集群不断成长壮大

　　牢固树立抓项目就是抓发展的理念。2010年，实施5000万元以上重点项目356个、总投资2780亿元，其中亿元以上161个、总投资2600亿元。

　　一批影响力大、牵动作用强的重大项目相继投产或开工。投资79亿元的绥电二期并网发电，绥中电厂成为东北最大火力发电厂；渤船重工第二个30万吨级船坞竣工使用，全市年造船能力达到400万吨；5万吨/年TDI满负荷生产；葫芦岛港吞吐量达到1000万吨；石河港完成投资3亿元，围堰1900米；青山水库开工建设，321米导流洞竣工，主坝基础盖板完成工程量的75%；经过多年努力，投资200亿元的1000万吨/年炼油已报国家发改委待批；投资1000亿元的6×100万千瓦徐大堡核电，国家已批准开展前期工作；投资26亿元亚洲最大的建昌雨润食品产业园开工建设，建兴高速公路全面启动，建昌革命老区“脱贫致富、通达大海”的期盼，已不再是梦想，即将变成现实！

　　3、县域经济发展态势强劲，民营经济快速增长

　　通过实施县域经济“三年倍增计划”，强化农业基础地位，社会主义新农村建设成效显著。2010年，粮食喜获大丰收，总产量109万吨，同比增长51%；肉蛋奶、水果和水产品产量分别达到68万吨、91万吨和55万吨，同比增长20%、17%和9%。省级设施蔬菜小区、标准化畜禽养殖小区达到8468个和490个，分别是2005年的52倍和33倍。农业产业化龙头企业289个、农民专业合作社934个，分别带动农户46万个和28万个。农资连锁销售网点499个，新农村现代流通服务网络基本建立。加强耕地资源保护，实现总量占补平衡；利用滩涂造地1.4万亩，新增建设用地7155亩，同比增长46%；治理水土流失面积25万亩，有效灌溉面积增加8.7万亩，连续14年荣获省农建“大禹杯”。

　　民营经济得到发展。2010年，民营经济增加值234亿元，同比增长14%，占全市地区生产总值的45%。规模以上民营企业达到500个，是2005年的3倍；全市有30个民营企业获“全省民营明星企业”荣誉称号，兴城市被评为“中国泳装名城”，民营经济已成为经济发展的生力军。

　　4、城区经济发展步伐加快，服务业成为新的经济增长点

　　实施城区经济“三年倍增计划”，现代服务业集聚效应初步显现。2010年，服务业增加值212亿元，同比增长12%。龙湾CBD以及沃尔玛、大润发、特易购“三大超市”启动建设。商品房销售面积259万平方米，是2005年的5倍；商品房销售额92亿元，是2005年的11.5倍。葫芦岛银行沈阳分行获得批复，盛京、浦发、民生3个股份制银行落户，绥中长丰、兴城长兴、连山国信3个村镇银行成立，全市银行、保险、证券等金融机构达64个；金融机构存款余额820亿元，贷款余额490亿元，分别比年初增长19%和12.6%，金融支撑作用明显增强。旅游业发展步伐加快。接待境内外游客1320万人次，同比增长22%，是2005年的3倍；旅游业总收入实现92亿元，是2005年的4.3倍。

　　5、重点领域改革成绩显著，自主创新能力持续增强

坚持把改革创新作为推动经济发展、社会进步的强大动力和突破口。2010年，锦化集团完成破产重组，职工得到妥善安置，企业平稳运行，扭亏为盈。完成新一轮政府机构改革，稳妥推进事业单位机构分类和人事制度改革。深入实施农村综合改革、文化体制和公共事业改革，其他领域改革也取得积极成效。南票区及杨家杖子经济开发区被列入国家经济转型试点地区，进入了可持续发展的崭新阶段。积极“扩权强县”，绥中县成为全省享有市级经济社会管理权限第一县，提升了我市县域经济发展的竞争力。

　　坚持以科技创新引领产业结构升级。2010年，高新技术产品产值150亿元，同比增长71%。实施国家级、省级科技项目43个，申请发明专利92件，获得省科技进步奖3项。

　　6、生态宜居滨城建设加快，城乡面貌发生可喜变化

　　不断完善城市功能，提升城市品位，初步形成了连山、龙港、兴城“三位一体”大城区格局。龙湾CBD“一场五馆”和“901”人防工程主体基本完成；滨海北路、兴化路和化工大桥改造竣工；高铁新北站投入使用，成为展示城市形象的新窗口。实现城市综合执法一体化，拆除违章建筑5万平方米。1066个行政村实现“村村通”油路。五里河、连山河治理实现“治污、防洪、景观”三大目标，经受了60年一遇特大洪水考验。建成8个污水处理厂，乌金塘水库除钼净化工程竣工，恢复向城区达标供水。推进辽西绿色生态长廊和生态公园建设，获省“绿叶杯”十连冠。城市空气质量优良天数稳定在96%以上。单位GDP能耗和主要污染物SO2、COD减排，分别完成“十一五”任务的108%、129%和109%。

　　7、民生工程惠及千家万户，群众生活明显改善

　　始终把保障和改善民生作为各项工作的出发点和落脚点，倾情倾力为群众办实事、解难题。2010年，全市筹措资金53亿元，实施系列民生工程。一是新增实名制就业5.6万人，城镇登记失业率3.5%，保持零就业家庭动态管理为零。二是覆盖城乡的社会保障体系逐步完善，18万城乡低保群众实现“应保尽保”；建昌县在全省率先开展“新农保”试点，31.2万农民“老有所养”。三是落实惠农强农政策，发放惠农补贴3.7亿元。整村推进扶贫39个，5万人实现稳定脱贫。四是新建经济适用房1400套、廉租住房4725户，保障了困难群体住房需求。五是全部免除城乡义务教育阶段学杂费和28万农村学生课本费。六是改造新老城区供水管线120公里；建设农村饮水安全工程301处，12万群众受益。七是实施城市美化、亮化、绿化工程，城区11条主次干道告别“摸黑路”。八是综合整治城市客运交通，规范管理出租车，创新公交管理体制，彻底解决了中巴客车运营无序的“顽疾”，百辆新型公交车投入使用，滨城又增添了一道靓丽的风景线！

　　8、社会事业全面进步，精神文明建设深入开展

　　深入实施人才强市战略，加大对科技、教育、文化、卫生等社会事业的投入，推进基本公共服务均等化。2010年，引进海外研发团队10个、国外智力项目7个。九年一贯制（寄宿）学校达到71所，高考升学率达到86.7%，高等院校在校生达到2.6万人，渤海船院成为全国示范学校。文化事业得到发展，新编歌舞剧《虹螺女》在全省第八届艺术节上获得6项大奖；“辽西太平鼓”和“葫芦剪纸”在上海世博会精彩亮相。实施全民健身计划，竞技体育取得长足发展，我市两位选手获得亚运会金牌。公共卫生体系逐步健全，食品药品监管力度加大。气象事业得到加强，自然灾害监测预警能力显著提高。第六次全国人口普查工作全面展开。统筹推进人口和计划生育工作，人口出生率控制在6.88‰以内。实现民族团结、宗教和睦。市志工作取得新突破，填补了建市以来没有市志的空白。其他各项事业也取得明显进步。

　　积极推进学习型机关、文明村镇、文明社区和文明行业等精神文明创建活动。“送温暖、献爱心”，支援四川和青海灾后重建，捐款捐物2340万元。国防教育不断深入，双拥共建、民兵预备役和国家安全工作成效显著。

　　9、民主法制建设得到加强，依法行政能力明显提高

　　积极营造民主、和谐、安定的发展环境，政通人和的社会局面得到巩固和发展。自觉接受人大及其常委会的法律监督，坚持重大事项向人大报告制度，认真落实人大的各项决议、决定；主动接受政协民主监督，广泛听取人大代表、政协委员和民主党派、工商联、无党派人士的意见和建议，民主政治建设全面加强。2010年，接受人大视察、检查23次，向人大常委会报告工作20次；接受政协视察、调研12次，向政协通报工作3次；办理代表建议73件、政协提案409件，办复率100%，满意率99%以上。建立人大代表联络员制度和政协委员民主监督员制度，每月送阅《政府工作》简报，广泛征求人大代表、政协委员的意见和建议，实现与代表、委员联络常态化。完善听证制度，强化审计监督，设立政务公开网站，实行行政审批网上公开，增强了政府工作透明度和公信力。全面治理“三乱”，纠正部门和行业不正之风，反腐倡廉工作取得新成效。

　　积极创建“平安葫芦岛”，全面落实社会治安综合治理各项措施，严厉打击各类违法犯罪，道路交通整治和消防工作成效显著，社会火灾防控水平得到提高，人民群众安全感明显增强。深入开展“信访工作效率年”活动，矛盾纠纷排查机制不断健全。建立了突发公共事件应急管理体系。加强专项整治，安全生产形势总体平稳，荣获“省安全生产工作先进市”称号，连续四年被评为“全国安全生产月活动优秀单位”。

　　各位代表，刚刚过去的五年，我市经济社会发展取得的重大成就，是市委正确领导的结果，是市人大和市政协监督支持的结果，是全市广大干部群众团结奋斗的结果。在此，我代表市政府，向全市人民，向人大代表、政协委员，向各民主党派、工商联、无党派人士、人民团体，向驻葫部队指战员、武警官兵、公安民警和中省直驻在单位，向所有关心、支持葫芦岛建设和发展的各界朋友，表示崇高的敬意和衷心的感谢！

　　在看到成绩的同时，我们必须清醒地认识到，发展中还面临不少困难和问题：经济总量小，区域经济发展不平衡，民营经济占国民经济比重偏低；利用外资规模较小，缺少具有牵动作用的龙头项目；城镇化率低，城市功能不完善；就业和社会保障、社会稳定等工作压力较大。另外，政府自身建设有待进一步加强，软环境建设还存在薄弱环节。今后，我们必须本着对事业高度负责的精神，采取有力措施，认真解决这些问题，决不辜负全市人民的期望。

二、“十二五”奋斗目标和主要任务

　　各位代表，“十二五”时期，是葫芦岛实现赶超发展的重要战略机遇期。站在新的起跑线上，我们一定要倍加珍惜来之不易的良好发展局面，“不动摇、不懈怠”，确保高水平开局，高标准起步。

　　“十二五”时期，全市经济社会发展的指导思想是：以邓小平理论和“三个代表”重要思想为指导，全面贯彻落实科学发展观，紧紧抓住东北振兴和辽宁沿海经济带开发开放的历史机遇，以科学发展、创新发展、和谐发展为主题，以加快转变经济发展方式为主线，以富民强市、保障和改善民生为立足点，继续实施工业强市、项目支撑和沿海开发战略，深化改革开放，加强社会建设，保护生态环境，推动经济社会又好又快发展。

　　今后五年，主要奋斗目标和任务是：

　　——主要经济指标实现“倍增”。到2015年，地区生产总值、财政一般预算收入、社会消费品零售总额翻一番，分别达到1000亿元、120亿元和470亿元；全社会固定资产投资年均增长26%，实际利用外资年均增长30%。其他指标也要倍增发展。

　　——人民生活水平明显提高。坚持把保障和改善民生，放在更加突出的位置。五年新增就业岗位20万个，城镇登记失业率控制在4%以内。城镇居民人均可支配收入和农民人均纯收入均要实现翻番。

　　——城镇化进程进一步加快。以葫芦岛主城区和绥中滨海新城两个百平方公里大城市建设为核心，沿261公里海岸线，形成“城市带、产业带、旅游带和生态带”。城镇化率提高到50%。

　　——沿海经济带加速隆起。坚持“以港兴市”，加快港口群建设，促进沿海经济带大发展。每个县区至少建成1个产值超100亿元的产业园区，其中3个超300亿元。

　　——县区经济跨上新台阶。加快社会主义新农村建设步伐，实施新一轮“三年倍增计划”，构筑城乡共同发展新格局。大力发展旅游文化产业，到2015年，旅游总收入实现220亿元，年均增长20%，把旅游业培育成新的支柱产业。扩大县域和城区经济社会管理权限，支持绥中县进入“全国百强县”。

　　——社会更加和谐稳定。坚持公益性和普惠性，发展各项社会事业。构建覆盖城乡的公共服务体系，增强社会保障能力。加强民主法制和精神文明建设，营造公平、正义的社会环境。

　　各位代表，蓝图鼓舞斗志，信心凝聚力量。我们坚信，在市委的正确领导下，在市人大、市政协和社会各界的大力支持下，经过全市人民五年的团结奋斗，我市综合竞争力一定会显著增强，葫芦岛一定能够在辽宁沿海经济带上振翅腾飞，实现经济社会发展的新跨越！

三、2011年主要工作安排

　　各位代表，今年是实施“十二五”规划的开局之年。我们一定要咬住目标不放松，上半年“双过半”，全年实现“两个不低于”，做到开局之年首战告捷。

　　主要预期目标是：地区生产总值和财政一般预算收入分别增长15%，全社会固定资产投资和外贸出口分别增长30%以上，实际利用外资增长100%，社会消费品零售总额增长18%，城镇居民人均可支配收入、农民人均纯收入分别增长15%。这些都是必保目标，在实际工作中，我们要力争上游，向更高的目标努力。

　　今年，要重点抓好9个方面的工作：

　　1、推进沿海经济带建设

　　完善基础，强化招商。12个主题概念产业园区完成固定资产投资600亿元，增长100%，逐步形成年销售产值超百亿元的产业集群。创新重点园区管理体制和运行机制，增加园区发展活力。提升葫芦岛港开放水平，完成石河港通用泊位主体工程，启动兴城港建设。以港带区，以港兴市，加速“港区一体化”进程。同步推进疏港铁路和锡绥铁路建设，打造“辽冀蒙能源重要出海口”。

　　提高招商引资水平。“走出去、请进来”，深化区域经济合作，抓好产业集群招商，引进内资增长50%。实施“引进重大项目攻坚计划”，引进世界500强或行业内世界100强投资项目2个以上；每个省级重点园区都要争取新上投资2亿元以上项目30个。

　　2、加快重点项目建设

　　规划实施5000万元以上重点项目350个，总投资4354亿元。以更大的热情、更高的干劲，实施“项目牵动”战略，务必在行业旗舰项目建设上实现新突破。3万吨镍、6000吨钴、渤船核电装备、30万吨铝型材、15万吨电解铜、TDI二期等项目，都要开工建设。青山水库、猴山水库、建兴高速要加快建设速度。绥电三期、南电三期、大唐热电、国电热电等项目，全面启动前期工作。投资1000亿元的徐大堡核电、投资200亿元的1000万吨炼油等重大项目，已经列入全省重点项目，对我市未来发展具有战略意义；我们要举全市之力，加快推进速度，积聚发展后劲，让葫芦岛在辽宁老工业基地全面振兴中脱颖而出！

　　深入实施“工业五项工程”，加快重点产业集群和特色产业基地建设。实施海外并购工程，推动产业升级，培育一批高新技术企业，实现高新技术产品增加值增长20%以上。加强节能降耗和淘汰落后产能工作，确保万元工业增加值能耗下降5%、用水量下降4%以上。继续深化国有企业改革，妥善处理改制企业历史遗留问题，完成锦化集团破产重组后续工作。

　　3、发展县域和城区经济

　　加快社会主义新农村建设，启动县域经济新一轮“三年倍增计划”。推进农业产业化，新增农业产业化龙头企业50个、农民专业合作社116个。加快种植业、养殖业产业结构调整，新建省级设施蔬菜小区4300个、标准化畜牧养殖小区100个，建设前所“现代生态农业园区”。粮食总产量稳定在90万吨以上。抓好县域产业集群，做大兴城泳装品牌。深化“扩权强县”工作，确保绥中县按照新的管理体制机制高效运行；积极推进建昌雨润食品、汇源果蔬和嘉泰葡萄酒“三大项目”投产，建设亚洲最大的农副产品加工基地，全力扶持革命老区建昌脱贫致富奔小康！

　　全面完成城区经济“三年倍增计划”任务。服务业增加值增长15%。打出以“温泉和大海”为主题的旅游名片，加快兴城滨海温泉城建设，全面启动兴城古城和觉华岛开发，发展旅游文化产业，旅游总收入增长20%以上。推进龙湾CBD和连山、龙港“两大商贸集聚区”建设，进一步改善消费环境。规范房地产开发秩序，房地产开发面积580万平方米，增长14%。加大简政放权力度，增强城区经济发展活力。积极推进龙港区“全域城市化”，支持南票区建设国家煤炭储配基地；用好用足国家扶持政策，加快南票和杨家杖子地区产业转型步伐。

　　4、发展壮大民营经济

　　实施中小企业“成长工程”，鼓励和引导民营资本进入国家准许的所有领域，增强发展活力。加大信贷扶持力度，引进股权投资基金，破解中小企业融资难题。完善政策激励机制，推进企业上市。努力为民营企业“走出去”创造条件，帮助民营企业转型升级，做大做强。坚决治乱减负，改善营商环境，一定要在全市形成支持、服务民营经济发展的强大合力！

　　5、推进生态宜居滨城建设

　　进一步完善城市规划，以龙湾CBD为核心，加快高桥至曹庄的百平方公里沿海城市带和绥中滨海新城建设；规划建设人口超5万的滨海风情新市镇，优化沿海城镇布局。完善城市基础设施，实施锦葫路改造，完成兴化热源供热工程和污水处理厂提标改造工程。扩大城市美化亮化绿化范围，完成茨山河、月亮河治理。加强城市管理，改善市容市貌，整治市场秩序，提升城市品位。

　　发展循环经济和低碳经济。节约集约利用土地，积极通过填海和整理荒滩造地，用好“增减挂钩”政策，解决土地“瓶颈问题”。加强节能减排和环境保护工作，落实“三同时”制度，SO2和COD排放量分别下降1.5%。推进“辽西绿化生态长廊建设工程”，开展建设绿色村庄活动，全年植树造林68.5万亩；创新河道管理体制，下决心整治河流，修复生态，控制污染，保护水源。

　　6、全力抓好系列“民生工程”

　　继续巩固民生成果，实名制就业、普惠制就业培训和有组织劳务输出，分别新增4万人、4.5万人和5万人以上，确保零就业家庭动态管理为零。加强社会保障体系建设，提高企业离退休人员基本养老金、城乡低保和在乡复员军人生活补助标准。实施“整村脱贫行动计划”，推进70个整村扶贫项目，实现10万群众稳定脱贫。

　　今年，要集中力量抓好7件实事：一是建立全程资助、全面覆盖的助学保障政策体系，决不让城乡贫困家庭子女因贫困而失学。二是实施学前教育“三年行动计划”，调整学前教育布局，解决“入园难、入园贵”问题。三是提高“新农合”保障标准，最高支付限额达到当地农民人均纯收入的6倍。四是新建饮水安全工程280处，解决11万农村居民饮水安全问题；完成市区自来水管网改造，让居民喝上安全水、放心水。五是新增廉租住房3400户、建设经济适用住房300套，确保困难群体“住有所居”。六是优先发展城市公交，推进公共交通管理体制改革；坚决依法取缔中心城区营运三轮车，营造安全有序的交通环境。七是力争三年内完成市区“城中村”改造，彻底解决“城中村”道路泥泞、污水横流、垃圾成堆的问题，改善群众居住环境和住房条件，让滨城面貌焕然一新！

　　7、协调发展各项社会事业

　　强化科技创新和人才工作，提高科技进步对经济社会发展的贡献率。推进农村中小学布局调整和资源整合，探索“初中向县城集中，小学向中心乡镇集中”的办学模式；积极发展中等职业教育和高等教育。深化文化体制改革，促进文化事业全面繁荣和文化产业快速发展。加强城乡医疗卫生服务体系建设，提高社区和乡村卫生一体化管理覆盖率。开展全民健身活动，完成“一场五馆”建设，做好第十二届全运会分赛区筹备工作。全面做好人口和计划生育工作，人口出生率控制在7.1‰以内。提高新闻出版、广播电视工作水平。做好档案史志工作，关心支持老龄、妇女儿童、残疾人和红十字会工作，发展社会福利和慈善事业。

　　8、加强精神文明和民主法治建设

　　自觉接受人大及其常委会的法律和工作监督，坚持向人大报告工作制度，认真落实人大的各项决议、决定，保持与人大代表联系常态化。主动接受政协的民主监督，深化民主监督员制度。认真办理人大代表的议案、建议和政协提案。广泛征求各民主党派、工商联、无党派人士以及工会、共青团、妇联等人民团体的意见和建议，主动接受新闻舆论和社会公众监督。深入开展社会治安综合治理，依法打击各种犯罪活动；认真做好群众来信来访工作；强化安全生产责任制，抓好消防安全、交通安全和食品药品安全，建设“平安葫芦岛”。严厉打击扰乱市场秩序行为，保持物价稳定。完善应急管理体系建设，提高预防和处置突发公共事件的能力。着力构建现代人防体系，增强整体防护能力。加强国防教育，支持部队建设，巩固新时期军政军民关系，争创“全国双拥模范城”四连冠。

　　9、切实加强政府自身建设

　　各位代表，葫芦岛加速发展的美好画卷已经展开，迫切需要我们改善发展环境，加强政风建设，提高行政能力。要改进工作作风，满怀激情，敢于担当，奋发有为，建设务实政府；要提高工作效率，树立责任意识，简化办事程序，雷厉风行，建设高效政府；要强化廉洁自律，加强党风廉政建设，树正气，压歪风，做表率，建设廉洁政府；要严格依法行政，严肃查处工作中不作为、乱作为行为，营造风清气正的政务环境，建设人民满意政府。

　　各位代表，站在新起点，攀登新高峰！让我们在市委的坚强领导下，团结一心，奋力拼搏，肩负起时代赋予的崇高使命，用实际行动创造“十二五”新的辉煌！
